# Electrospinning Hetero-Nanofibers In_2_O_3_/SnO_2_ of Homotype Heterojunction with High Gas Sensing Activity

**DOI:** 10.3390/s17081822

**Published:** 2017-08-09

**Authors:** Haiying Du, PengJun Yao, Yanhui Sun, Jing Wang, Huisheng Wang, Naisen Yu

**Affiliations:** 1 College of mechanical and Electronic Engineering, Dalian Minzu University, Dalian 116600, China; duhaiying@dlnu.edu.cn; 2 School of Electronic Science and Technology, Dalian University of Technology, Dalian 116023, China; wangjing@dlut.edu.cn (J.W.); 13204115731@163.com (H.W.); 3 Department of Electrical and Computer Engineering, College of Engineering, Iowa State University, Ames, IA 50011, USA; 4 School of Educational Technology, Shenyang Normal University, Shenyang 110034, China; yaopj@synu.edu.cn; 5 School of Physics and Materials Engineering, Dalian Minzu University, Dalian 116600, China; yunaisen@dlnu.edu.cn

**Keywords:** electrospinning, composite hetero-nanofibers, homotype heterojunction, indoor volatile organic gases, gas sensing properties, gas sensing mechanism

## Abstract

In_2_O_3_/SnO_2_ composite hetero-nanofibers were synthesized by an electrospinning technique for detecting indoor volatile organic gases. The physical and chemical properties of In_2_O_3_/SnO_2_ hetero-nanofibers were characterized and analyzed by X-ray diffraction (XRD), field emission scanning electron microscope (FE-SEM), Energy Dispersive X-Ray Spectroscopy (EDX), specific surface Brunauer–Emmett–Teller (BET) and X-ray photoelectron spectroscopy (XPS). Gas sensing properties of In_2_O_3_/SnO_2_ composite hetero-nanofibers were measured with six kinds of indoor volatile organic gases in concentration range of 0.5~50 ppm at the operating temperature of 275 °C. The In_2_O_3_/SnO_2_ composite hetero-nanofibers sensor exhibited good formaldehyde sensing properties, which would be attributed to the formation of n-n homotype heterojunction in the In_2_O_3_/SnO_2_ composite hetero-nanofibers. Finally, the sensing mechanism of the In_2_O_3_/SnO_2_ composite hetero-nanofibers was analyzed based on the energy-band principle.

## 1. Introduction

With the aggravation of environmental pollution and the deterioration of the climate, human beings have put forward higher requirements on the gas detection of living environment. Semiconductor metal oxides have been investigated extensively and applied widely in the various fields of gas detection technology for its low cost, high accuracy, small size and easy to carry [1,2,3,4]. There are many parameters and factors that affect and limit gas sensor applications, such as adsorption ability, catalytic activity, sensitivity, selectivity, thermodynamic stability, and so on [5]. 

Among the various semiconductor gas sensitive materials, SnO_2_ is used popularly as gas sensors because of excellent adsorption ability, high sensitivity at relatively low operating temperatures, but there is a challenge to improve the sensing performance due to the cross-sensitivity of SnO_2_ gas sensors [6,7]. To enhance the gas sensing properties of SnO_2_, doping or surface modification methods have been adopted [8,9]. In addition, the preparation of composite semiconductor metal oxides attracts more attention [10,11]. The physical interface between two dissimilar materials is often referred to as a heterojunction. The new composite incorporates two components forming a heterostructure. Gas sensors based on the composite are more sensitive than the individual components due to the interface heterostructure and a synergistic effect between the two components [5,12]. Some researchers found that the heterojunction acts as a lever in electron transfer, through which electron transport is facilitated or restrained resulting in superior sensing performance [13,14,15,16,17,18]. Recently, many heterostructure composites such as SnO_2_/ZnO [19,20], TiO_2_/SnO_2_ [21], SnO_2_/Fe_2_O_3_ [22] have been prepared to be applied as gas sensing materials to obtain the sensitive and selective gas sensors. In_2_O_3_ and SnO_2_ heterostructure composites have also been synthesized to enhance the properties of sensors [23,24,25]. In_2_O_3_/SnO_2_ heterojunction microstructures synthesized by a facile room temperature solid-state reaction route exhibited high response to chlorine [26]. A rapidly responding methanol sensor based on electrospinning In_2_O_3_/SnO_2_ nanofibers was reported [27]. Many researchers have found that In_2_O_3_ can be used to effectively limit grain growth of the SnO_2_ host nanoparticles in the high temperature synthesis process to affect gas sensitivity and selectivity [28]. However, to the best of our knowledge, few reports have shown that In_2_O_3_–SnO_2_ heterostructure composites have an excellent response and selectivity for formaldehyde.

In this manuscript, In_2_O_3_/SnO_2_ composite hetero-nanofibers with n-n-type heterojunction were synthesized successfully by a mixed electrospinning method. Gas sensors based on In_2_O_3_/SnO_2_ composite hetero-nanofibers were fabricated for detecting low concentration indoor volatile organic gas. Six kinds of indoor volatile organic gases (including formaldehyde, ethanol, acetone, methanol, toluene and ammonia) were tested based on In_2_O_3_/SnO_2_ composite hetero-nanofibers sensors by a static gas test method. It was found that In_2_O_3_/SnO_2_ composite hetero-nanofibers sensors show excellent formaldehyde sensing properties superior to the pure SnO_2_ or In_2_O_3_ nanofibers sensors at a low operating temperature of 275 °C, which could be attributed to the formation of n-n homotype heterojunction in the In_2_O_3_/SnO_2_ composite hetero-nanofibers. 

## 2. Experimental

### 2.1. Preparation and Characterization of In_2_O_3_/SnO_2_ Composite Hetero-Nanofibers

All chemical reagents used were of the analytical grade in the experiments; 0.76 g indium nitrate (In(NO_3_)_3_·4.5H_2_O), 0.45 g stannous chloride (SnCl_2_·2H_2_O) and 10 ml ethanol were mixed and stirred vigorously for 1 h. The molar ratio of In to Sn in the composites is 1:1. Then, 1 g Polyvinyl pyrrolidone (PVP, M_w_ = 1,300,000) and 8 ml N, N Dimethylformamide (DMF) were added to the mixture solution and stirred for 3 h until dissolved evenly at the room temperature. The colorless and transparent mixed electrospinning precursor solution was formed.

The mixed precursor solution was loaded into the glass syringe with a metal jet. A high DC voltage was applied between the jet and the collector. The precursor was ejected from the jet and formed a wet nanofibers net under the electric force. Then, the net was heated at 600 °C for 120 min in the air using a muffle furnace with temperature control system. PVP and water in the polymer nanofibers volatilized during the annealing process. Finally, the light yellow In_2_O_3_/SnO_2_ composite hetero-nanofibers were obtained. Pure SnO_2_ and In_2_O_3_ electrospinning nanofibers were synthesized by using a traditional electrospinning device, respectively. The annealing process of SnO_2_ and In_2_O_3_ electrospinning nanofibers were the same as that of In_2_O_3_/SnO_2_ composite hetero-nanofibers. 

The phase structure and morphology of the In_2_O_3_/SnO_2_ composite hetero-nanofibers were characterized by an X-ray diffraction instrument (XRD: D/Max 2400, Rigaku, Japan) in 2*θ* region of 20~80° with Cu *Kα*1 radiation and a field emission scanning electron microscope (FE-SEM: Hitachi S-4800, Japan), respectively. The composition and contents were analyzed by using an Energy Dispersive X-Ray Spectroscopy (FE-SEM: Hitachi S-4800, Japan). The specific surface was measured by a Brunauer–Emmett–Teller (BET: Quanta AUTOSORB-1-MP, Boynton Beach, FL, USA) method. The chemical composition and surface state were examined using high-resolution X-ray photoelectron spectroscopy (XPS) on a spectrometer (XPS: Thermo ESCALAB 250Xi, Westmont, IL, USA). The AC impedance spectroscopy of electrochemical properties was carried out and analyzed on an impedance analyzer (B1500A, Agilent, Santa Clara, CA, USA).

### 2.2. Sensors Fabrication and Measurements

**Figure 1:**
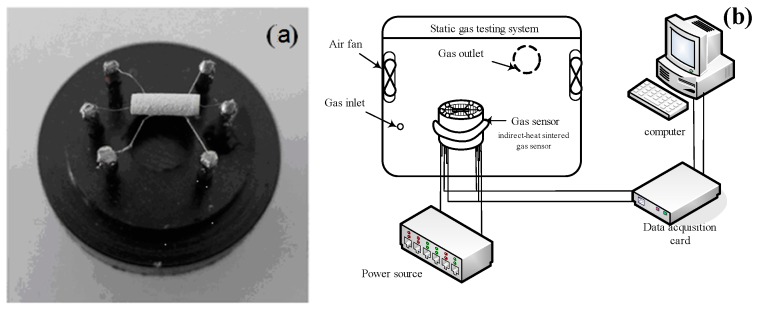
(**a**) The picture of inside-heated gas sensor; (**b**) The structure schematic diagram of the static gas testing system.

In_2_O_3_/SnO_2_ composite hetero-nanofibers were mixed with deionized water to form a paste. The paste was coated onto the surface of a ceramic tube with a pair of gold electrodes to form a sensitive film (about 300 μm thick) and dried in hot air, then, annealed at 500 °C for 2 h in open air. Finally, a Ni-Cr heating wire used as a heating electrode was inserted into the ceramic tube to form an inside-heated gas sensor. The inside-heated gas sensor picture is shown in Figure 1a. Indoor volatile organic gases were measured based on In_2_O_3_/SnO_2_ composite hetero-nanofibers sensor using a static-state gas-sensing test method [17]. In the detection process of volatile organic gases, a given amount of target gas was injected into a hermetic chamber (50 L in volume) by a syringe through a rubber plug. For a required concentration, the volume of the injected gas (*V*) can be calculated by theEquation (1) [29]:

(1) $$V=\frac{50\times C}{v\%}$$

where *C* is the concentration of the target gas (ppm), and *v*% is the volume fraction of bottled gas injected from the gas inlet hole. The gas response (*S*) was defined as a ratio of the electrical resistance of the sensor in the air (*R*_a_) to that in target gas (*R*_g_): *S* = *R*_a_/*R*_g_ [17]. A structure schematic diagram of the static gas testing system was shown in Figure 1b.

## 3. Results and Discussion

### 3.1. Characterization

**Figure 2:**
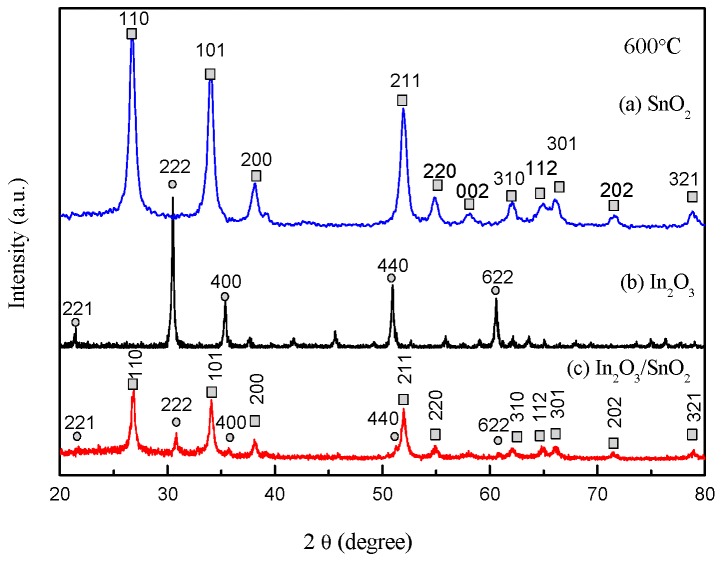
XRD patterns of (**a**) SnO_2_, (**b**) In_3_O_2_ and (**c**) In_2_O_3_/SnO_2_ composite hetero-nanofibers.

The XRD patterns of the pure SnO_2_, In_2_O_3_ nanofibers and In_2_O_3_/SnO_2_ composite hetero-nanofibers are shown in Figure 2a–c. We can see from Figure 2a,b that the SnO_2_ spinning nanofibers belong to tetragonal rutile [Joint Committee on Powder Diffraction Standards [(JCPDS) 41-1445] (square) and the In_2_O_3_ nanofibers belong to cubic phase [JCPDS 71-2195] (circle). As shown in Figure 2c, both SnO_2_ and In_2_O_3_ exist in the In_2_O_3_/SnO_2_ composite hetero-nanofibers. According to the results calculating by the Debye–Scherrer equation [30]:

(2) $$\tau =\frac{K\lambda }{\beta \cos\theta }$$

where τ is the size of the crystalline domains, which may be smaller or equal to the grain size; *K* is the dimensionless shape factor, with a value close to unity. The shape factor has a typical value of about 0.9; *λ* is the X-ray wavelength; *β* is the line broadening at half the maximum intensity, after subtracting the instrumental line broadening, in radians. This quantity is also sometimes denoted as Δ(2*θ*); *θ* is the Bragg angle. The average crystallite sizes of the pure SnO_2_ nanofibers (Figure 2a) and the pure In_2_O_3_ nanofibers (Figure 2b) are 21 nm and 37 nm, respectively. Meanwhile, the average crystallite size of the In_2_O_3_/SnO_2_ composite hetero-nanofibers (Figure 2c) is 20 nm. The average crystallite size of In_2_O_3_/SnO_2_ composite hetero-nanofibers is much smaller than that of pure In_2_O_3_ nanofibers and the same as that of the pure SnO_2_ nanofibers.

**Figure 3:**
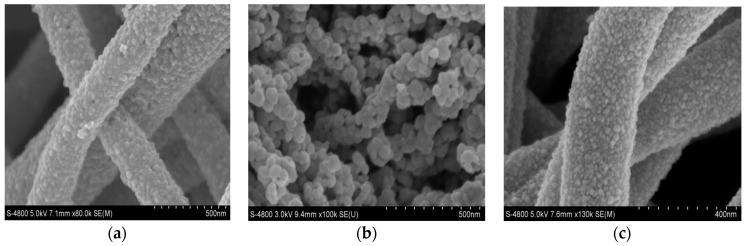
SEM images of (**a**) SnO_2_ nanofibers, (**b**) In_2_O_3_ nanofibers, (**c**) In_2_O_3_/SnO_2_ composite hetero-nanofibers.

Figure 3a–c give the SEM images of the as-prepared SnO_2_ nanofibers, In_2_O_3_ nanofibers and In_2_O_3_/SnO_2_ composite hetero-nanofibers, respectively. It can be seen from Figure 3a–c that SnO_2_, In_2_O_3_ and In_2_O_3_/SnO_2_ nanofibers are hierarchical structures composed of small nanoparticles. As depicted in Figure 3a, the diameters of SnO_2_ nanofibers are ~200 nm, and the diameters of SnO_2_ nanoparticles are about 20 nm. Figure 3b shows that the surfaces of the In_2_O_3_ nanofibers are irregular and slightly rough with the diameters of 150 nm. Each In_2_O_3_ nanofiber is composed of many nanoparticles with the diameters of 35–40 nm. Figure 3c displays that the diameters of In_2_O_3_/SnO_2_ composite hetero-nanofibers are about 410 nm. The In_2_O_3_/SnO_2_ nanofibers are much thicker than SnO_2_ and In_2_O_3_ nanofibers. The diameters of In_2_O_3_/SnO_2_ nanoparticles are about 16 nm. The diameters of In_2_O_3_/SnO_2_ nanoparticles are smaller than those of In_2_O_3_ or SnO_2_ nanofibers. 

There is a big difference among the morphologies and structures of the three kinds of nanofibers. In the process of nanofibers formation, the forces of each nanoparticle will be influenced by many factors, such as the surface tension, solubility, electric field force and so on. Of the factors that affect the morphology and structures, the conductivity of salt solutions must be considered. The conductivities of SnCl_2_ solution, In(NO_3_)_3_ solution and the mixed solution are different, which will result in the great difference of the electric field force and the Coulomb force of the nanofibers ejected from the spinning jet. These will have an effect on the diameters and morphologies of the nanofibers. At the same time, SnCl_2_ and In(NO_3_)_3_ are mixed sufficiently, the distribution of Sn and In ions in the solution is relative uniformity. The arrangement and distribution of two kinds of nanoparticles in the nanofibers are uniform.

**Figure 4:**
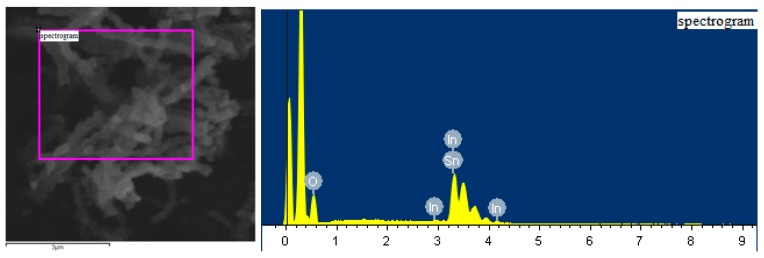
EDX spectra of In_3_O_2_/SnO_2_ composite hetero-nanofibers.

Figure 4 gives the Energy Dispersive X-ray (EDX) spectra of the In_2_O_3_/SnO_2_ composite hetero-nanofibers. It can be observed in Figure 4 that O, In, and Sn elements are present in the spectra of the In_2_O_3_/SnO_2_ composite hetero-nanofibers. Table 1 lists the elemental contents of O, In and Sn in In_2_O_3_/SnO_2_ composite. The weight percentages of O, In and Sn are 31.5%, 19.6% and 48.9%, respectively. The atomic percentages of O, In and Sn are 77.2%, 6.7% and 16.1%, respectively. 

**Figure 5:**
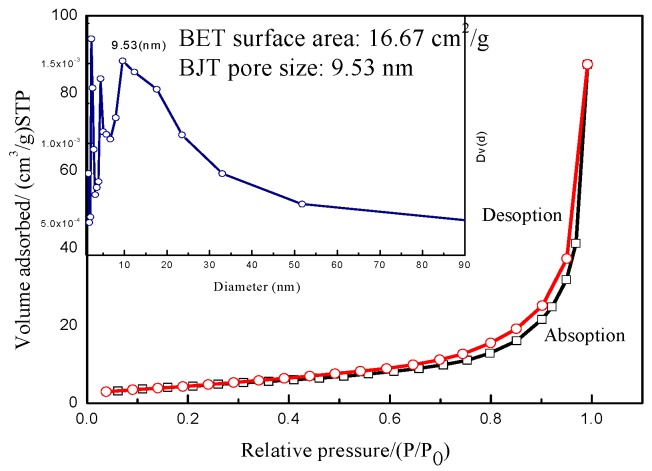
N_2_ isotherms adsorption–desorption curves of In_2_O_3_/SnO_2_ composite hetero-nanofibers.

Figure 5 gives the N_2_ isotherms adsorption–desorption curves of In_2_O_3_/SnO_2_ composite hetero-nanofibers. As shown in Figure 5, the N_2_ isotherms adsorption–desorption curves of In_2_O_3_/SnO_2_ composite hetero-nanofibers belong to the IV-type isotherms, which demonstrates that In_2_O_3_/SnO_2_ composite nanofibers are the mesoporous solid materials. The adsorption curve and desorption curve are different. H3-type hysteresis loop is shown in Figure 5, which means that the whole adsorption process can be divided into the monolayer adsorption process of the first zone, the multilayer adsorption process of the second zone, and the capillary condensation process of the third zone. The adsorption process belongs to the weak saturation adsorption [31]. According to the inset in Figure 5, the specific surface (BET) of In_2_O_3_/SnO_2_ composite hetero-nanofibers is 16.67 m^2^/g, and the pore distribution is in the range of 2.3–9.5 nm. The pore distribution of In_2_O_3_/SnO_2_ composite hetero-nanofibers spreads out widely and disorderly. The saturated zone is not obvious. The results show that the In_2_O_3_/SnO_2_ composite hetero-nanofibers adsorption mechanism is disorder mesoporous adsorption. This kind of hierarchically composite fibers composed of small nanoparticles does not show significant absorption limit in the relatively high-pressure region.

**Figure 6:**
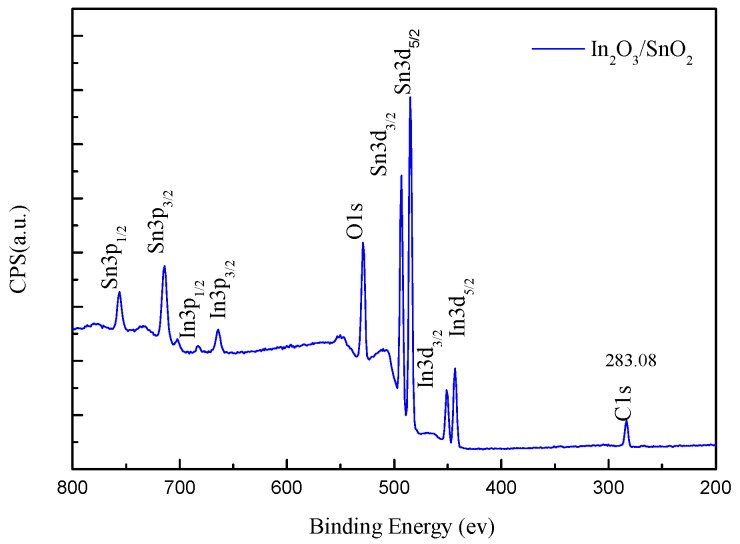
XPS survey spectrum of In_2_O_3_/SnO_2_ nanofibers.

To further confirm the chemical composition and surface oxidation state, the X-ray photoelectron spectroscopy (XPS) of In_2_O_3_/SnO_2_ composite hetero-nanofibers is performed, and the XPS survey spectrum of In_2_O_3_/SnO_2_ composite hetero-nanofibers is shown in Figure 6. As expected, the XPS spectrum of In_2_O_3_/SnO_2_ is dominated by the lines of In, Sn, O and C. The C element is attributed to adventitious carbon-based additives and the C1s, whose banding energy peak locating at 283.08 eV, is used as reference for calibration. There are nine main peaks in the spectrum of In_2_O_3_/SnO_2_ composite hetero-nanofibers, and they are Sn3p_1/2_, Sn3p_3/2_, In3p_1/2_, In3p_3/2_, O1s, Sn3d_3/2_, Sn3d_5/2_, In3d_3/2_ and In3d_5/2_, respectively. 

**Figure 7:**
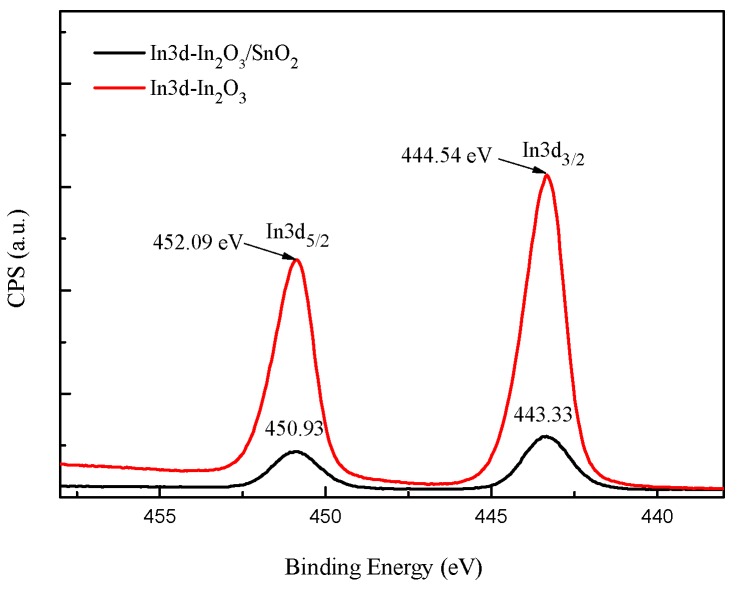
In3d core level XPS spectrum of In_2_O_3_ nanofibers and In_2_O_3_/SnO_2_ composite hetero-nanofibers.

**Figure 8:**
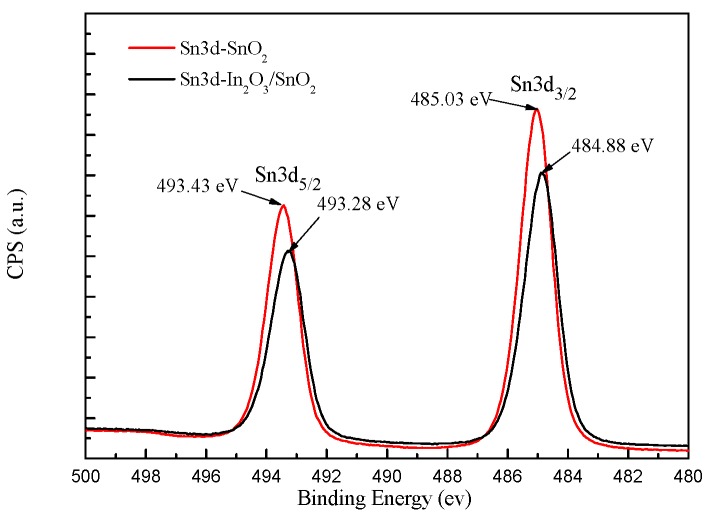
Sn3d core level XPS spectrum of SnO_2_ nanofibers and In_2_O_3_/SnO_2_ composite hetero-nanofibers.

As can be seen in In3d core level XPS spectrum of In_2_O_3_/SnO_2_ composite hetero-nanofibers (Figure 7), two binding energy peaks centered at 443.33 eV and 450.93 eV are ascribed to In3d_5/2_ and In3d_3/2_, respectively, which originate from In-O in In_2_O_3_ lattice [32]. Figure 8 shows Sn3d core level XPS spectrum of In_2_O_3_/SnO_2_ composite hetero-nanofibers. As shown in Figure 8, the binding energy of Sn3d_3/2_ and Sn3d_5/2_ obtained from the XPS measurements are 484.88 eV and 493.28 eV, respectively, which indicates that Sn in the composites has s valence of +4 [33]. Compared with In3d_5/2_ (452.09 eV) and In3d_3/2_ (444.54 eV) of pure In_2_O_3_, the binding energy of both In3d_5/2_ and In3d_3/2_ of In_2_O_3_/SnO_2_ composite hetero-nanofibers moves to higher binding energy direction. On the contrary, the binding energy of both Sn3d_3/2_ and Sn3d_5/2_ of In_2_O_3_/SnO_2_ composite hetero-nanofibers moves to lower binding energy direction compared with Sn3d_3/2_ (493.43 eV) and Sn3d_5/2_ (485.03 eV) of pure SnO_2_ nanofibers. This phenomenon, due to electron transfer, will occur from In_2_O_3_ to SnO_2_ until the energy-band diagram of the n-n heterojunction of In_2_O_3_/SnO_2_ comes to equilibrium [14,34,35]. This phenomenon can also be explained by the strong interaction between In_2_O_3_ and SnO_2_, which will lead to the decreased surface activity of SnO_2_ and the increased surface activity of In_2_O_3_ [36].

**Figure 9:**
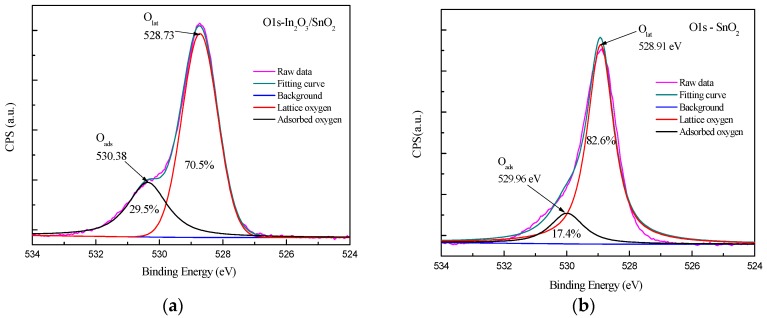

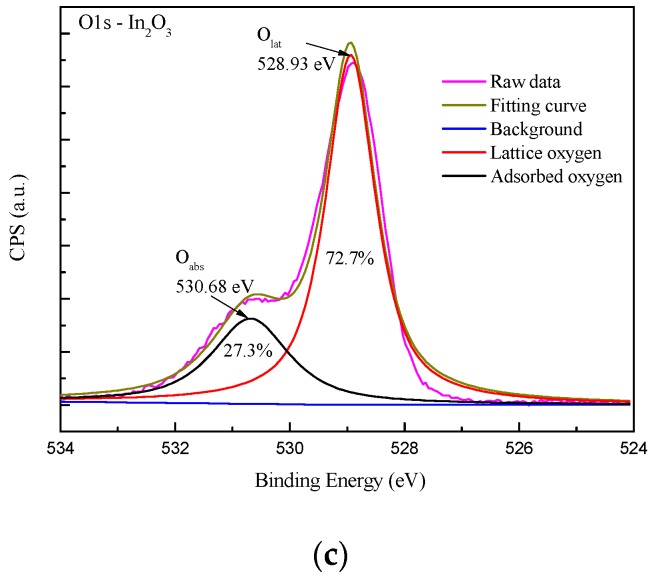
O1s core level XPS spectrum of (**a**) In_2_O_3_/SnO_2_ composite hetero-nanofibers (**b**) SnO_2_ nanofibers and (**c**) In_2_O_3_ nanofibers.

Figure 9 gives O1s core level XPS spectrums of In_2_O_3_/SnO_2_ composite hetero-nanofibers, In_2_O_3_ nanofibers and SnO_2_ nanofibers, respectively. As can be seen in Figure 9a, an asymmetric peak at 528.73 eV is shown on O1s core level XPS spectrum of In_2_O_3_/SnO_2_ composite nanofibers. The peak of O1s can be separated into two peaks at O_lat_ (528.73 eV) and O_ads_ (530.28). The O_lat_ (528.73 eV) can be assigned to the lattice oxygen on the surface of In_2_O_3_/SnO_2_ composite hetero-nanofibers. It will not participate in the chemical reaction in the process of gas adsorption below 300 °C. The O_ads_ is attributed to the adsorbed oxygen of In_2_O_3_/SnO_2_ composite hetero-nanofibers. Adsorbed oxygen participates in the reaction with activity in the process of gas adsorption. The relative intensities of O_lat_ and O_ads_ contributions are 70.5% and 29.5%, respectively. Similarly, the relative intensities of O_lat_ and O_ads_ contributions in SnO_2_ nanofibers are 82.6% and 17.4%, respectively (shown in Figure 9b). The relative intensities of O_lat_ and O_ads_ contributions in In_2_O_3_ nanofibers are 72.7% and 27.3%, respectively (shown in Figure 9c). The proportion of adsorbed oxygen in In_2_O_3_/SnO_2_ composite hetero-nanofibers is higher than that in pure SnO_2_ nanofibers or pure In_2_O_3_ nanofibers. It may improve adsorption capacity of In_2_O_3_/SnO_2_ composite hetero-nanofibers, and then, increase the response sensitivity of gas sensors [37,38,39]. 

### 3.2. Gas Sensing Properties

**Figure 10:**
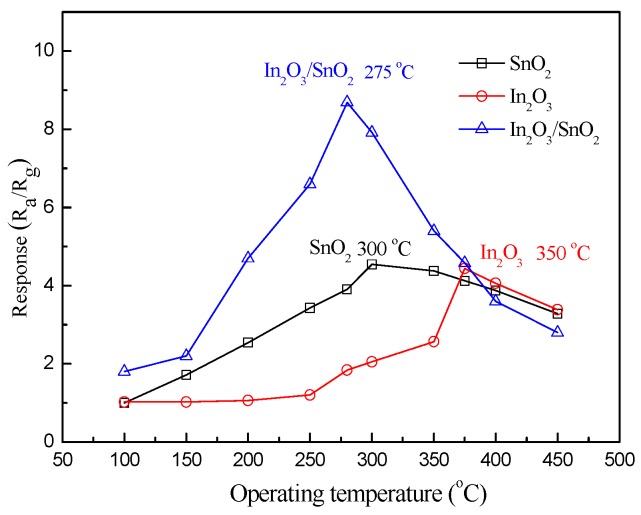
Responses of the SnO_2_, In_2_O_3_ and In_2_O_3_/SnO_2_ sensors as a function of operating temperature.

Figure 10 gives the response curves of the gas sensors based on SnO_2_, In_2_O_3_ and In_2_O_3_/SnO_2_ composite hetero-nanofibers versus operating temperatures to 10 ppm formaldehyde, respectively. It can be seen that the response of In_2_O_3_/SnO_2_ sensor increases as the operating temperature increases from 100 °C to 275 °C. The response of the gas sensor decreases when the operating temperature increases from 275 °C to 450 °C. The response reaches the maximum value (8.7) in 10 ppm formaldehyde at the operating temperature of 275 °C. Therefore, 275 °C is chosen as the optimum operating temperature of the In_2_O_3_/SnO_2_ composite hetero-nanofibers sensors. Similarly, 300 °C and 350 °C are selected as the optimum operating temperatures of the pure SnO_2_ and In_2_O_3_ nanofibers sensors, respectively. 

The optimum operating temperature of the In_2_O_3_/SnO_2_ composite hetero-nanofibers sensors is lower than that of pure SnO_2_ or In_2_O_3_ nanofibers sensors, which may be explained as follows. Comparing with the pristine In_2_O_3_ and SnO_2_ nanofibers, In_2_O_3_/SnO_2_ composite hetero-nanofibers have a different crystallographic structure, which is decided by their own combination of crystallographic planes, nanocrystal framing and structure, which is decided by its own total combination of surface electron parameters. It includes surface state density; energetic position of the levels, induced by adsorbed species, adsorption–desorption energies of interacted gas molecules, concentration of adsorption surface states, position of surface Fermi level, activation energy of native point defects, and so on. Also, the gas sensitivity, operating temperature and rate of response and recovery time are magnitude by combination of surface electron parameters according to the chemisorption theory of Volkenshtein [40,41,42,43], another reason maybe arises by transport characteristics of In_2_O_3_/SnO_2_ hetero-nanostructures. The electron transport characteristics of In_2_O_3_/SnO_2_ composite hetero-nanofibers will be significantly strongly tuned by the heterojunction barrier comparing the pristine In_2_O_3_ or SnO_2_ due to the formation of many semiconductor heterojunctions at interfaces. The conductivity of the heterojunctions will consequently be greatly increased. Therefore, the barrier with adjustable height controls the transport of electrons in the heterostructures, and accordingly controls the sensing characteristics of the nanocomposites, including the lower operating temperature [22].

**Figure 11:**
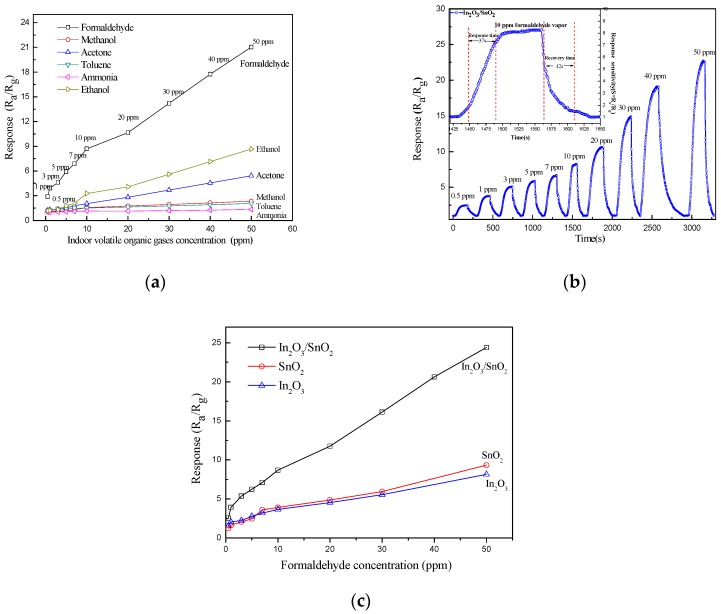
The gas sensing properties of the In_2_O_3_/SnO_2_ composite hetero-nanofibers sensor. (**a**) The relationship of response of In_2_O_3_/SnO_2_ gas sensor versus concentration of formaldehyde, methanol, acetone, toluene, ammonia and ethanol vapor. (**b**) The transient response curves of In_2_O_3_/SnO_2_ sensor to formaldehyde vapor with the concentration of 0.5–50 ppm. (**c**) The response of SnO_2_, In_2_O_3_ and In_2_O_3_/SnO_2_ sensors versus the concentration of formaldehyde vapor.

Figure 11a–c shows the gas sensing properties of the In_2_O_3_/SnO_2_ composite hetero-nanofibers sensor to indoor volatile organic gases (including formaldehyde, methanol, acetone, toluene, ammonia and ethanol) in the concentration range of 0.5–50 ppm at an operating temperature of 275 °C with relative humidity of 40% RH. Figure 11a shows the relationship of the response of In_2_O_3_/SnO_2_ sensor versus the concentration of formaldehyde, methanol, acetone, toluene, ammonia, ethanol vapor. The In_2_O_3_/SnO_2_ sensor shows the highest response to the formaldehyde vapor in the whole concentration range. The response to ethanol is lower than that to formaldehyde, and the response to ammonia is the lowest.

Figure 11b gives the transient response curves of the In_2_O_3_/SnO_2_ sensor to formaldehyde in the concentration of 0.5–50 ppm. In the experiment, ten cycles were recorded. The In_2_O_3_/SnO_2_ sensor shows excellent sensitivities to formaldehyde vapor. The inset shows the response and recovery time for 10 ppm formaldehyde vapor at an operating temperature of 275 °C with the relative humidity of 40% RH. It can be seen that the response and recovery time are 37 s and 42 s to 10 ppm formaldehyde vapor, respectively. 

In order to compare the gas sensing properties of In_2_O_3_/SnO_2_ composite hetero-nanofibers sensors with that of the SnO_2_ or In_2_O_3_ sensors, Figure 11c shows the response of SnO_2_, In_2_O_3_ and In_2_O_3_/SnO_2_ sensors versus the concentration of formaldehyde vapor at the operating temperature of 275 °C, respectively. The response curves of SnO_2_, In_2_O_3_ and In_2_O_3_/SnO_2_ sensors to formaldehyde vapor are basically linear when the formaldehyde vapor concentration is in the range of 0.5–50 ppm, respectively. The response of the In_2_O_3_/SnO_2_ composite hetero-nanofibers sensor to formaldehyde vapor is much higher than that of the SnO_2_ or In_2_O_3_ sensors. The response of the In_2_O_3_/SnO_2_ sensor to 50 ppm formaldehyde vapor is 24.4. The lowest concentration of formaldehyde detected by the In_2_O_3_/SnO_2_ sensor is 0.5 ppm with a response of 2.5.

### 3.3. The Gas Sensing Mechanism of the In_2_O_3_/SnO_2_ Composite Hetero-Nanofibers Sensor

**Figure 12:**
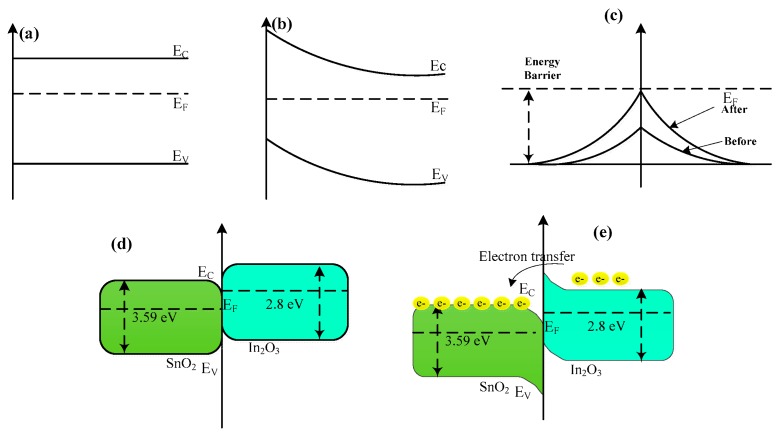
Energy-band diagram of the In_2_O_3_/SnO_2_ composite hetero-nanofibers system. (**a**) Energy-band of SnO_2_ before oxygen adsorption. (**b**) Energy-band of SnO_2_ after oxygen adsorption. (**c**) Energy-band change of SnO_2_ in energy barrier induced by oxygen adsorption. (**d**) Energy band of In_2_O_3_/SnO_2_ composite hetero-nanofibers system before oxygen adsorption. (**e**) Energy band of In_2_O_3_/SnO_2_ composite hetero-nanofibers system after oxygen adsorption.

The experiment results demonstrate that the In_2_O_3_/SnO_2_ sensors show the excellent formaldehyde sensing properties compared to those of SnO_2_ or In_2_O_3_ sensors, which means that the adsorption capability of In_2_O_3_/SnO_2_ composite hetero-nanofibers sensor to formaldehyde is greatly enhanced. The reason may be explained by the mechanism of oxygen adsorption. It is well known that the reducing gas will interact with the adsorbed oxygen on the surface of the semiconductor, which will lead to the change of sensor resistance. The more surface-adsorbed oxygen exists on the gas sensing material, the greater the sensor’s resistance changes when meeting the reducing volatile organic gases. Figure 12 illustrates the energy-band diagram of the In_2_O_3_/SnO_2_ composite hetero-nanofibers system. As seen in Figure 12a–c, the pre-adsorbed oxygen could give rise to a depletion layer near to the surface of n-type semiconducting oxides [44], which results in a band bending around the surface. It is well known that SnO_2_ and In_2_O_3_ are the prototypical n-type transparent conducting oxide semiconductor. The band gap and the work function of SnO_2_ are 3.59 eV and 4.9 eV, respectively [45], while the band gap and the work function of In_2_O_3_ are 2.8 eV and 5.28 eV, respectively. SnO_2_ has higher electron affinity (4.5 eV) than In_2_O_3_ (4.45 eV). Both the bottom of the conduction band and the top of the valence band of In_2_O_3_ are higher than those of SnO_2_ (Figure 12d,e), since the SnO_2_ has a lower work function and a strong electron affinity [46]. The transport of electrons should transport form In_2_O_3_ to SnO_2_ to overcome the heterojunction barriers [22,47]. When the In_2_O_3_/SnO_2_ heterostructures are exposed to reducing gases, the reaction between the adsorbed oxygen species and the reducing gases leads to the release of the trapped electrons back simultaneously into the conduction bands of the In_2_O_3_ and SnO_2_, resulting in a decrease in the width and height of the barrier potential at their interfaces, as shown in Figure 12e. Therefore, the conductivity of the heterojunction will consequently be greatly increased, the barrier with adjustable height controls the transport of electrons in the heterostructures, which will improve the gas sensing properties of In_2_O_3_/SnO_2_ composite hetero-nanofibers sensor [12,13,46,47].

**Figure 13:**
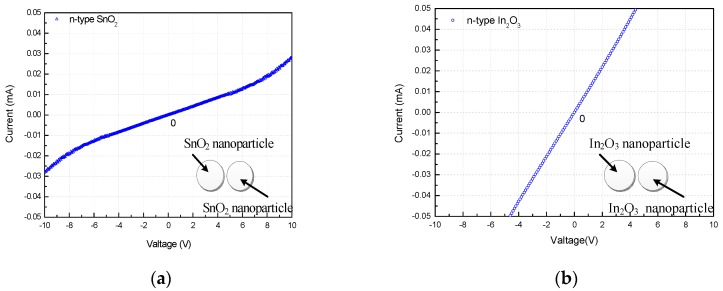

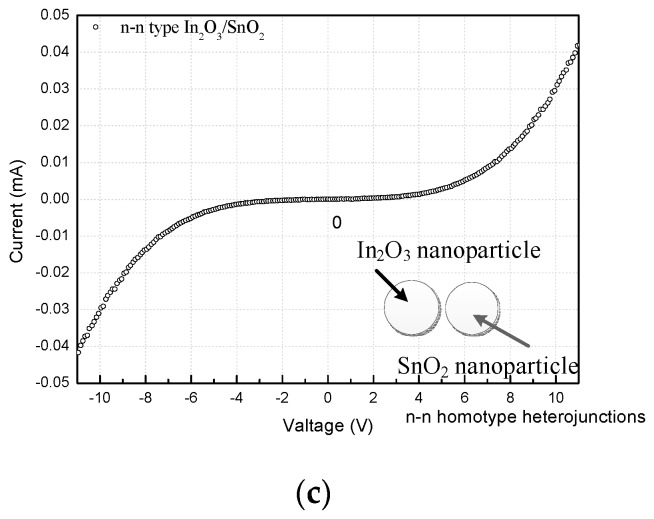
I-V curves of (**a**) SnO_2_, (**b**) In_2_O_3_ and (**c**) In_2_O_3_/SnO_2_ composite hetero-nanofibers.

In order to confirm that *n*-*n* homotype heterojunctions exist in In_2_O_3_/SnO_2_ composite hetero-nanofibers, electrochemical characteristics of SnO_2_, In_2_O_3_ and In_2_O_3_/SnO_2_ sensors were measured at the operating temperature of 275 °C. Figure 13 reports the double-isotype I-V characteristics of the SnO_2_, In_2_O_3_ and In_2_O_3_/SnO_2_ sensors, respectively. We can see that I-V curves of SnO_2_, In_2_O_3_ nanofibers are almost linear, which indicates that there is a good ohmic contact existing inside of the *n*-type SnO_2_ and In_2_O_3_ sensors, respectively. Meanwhile, the I-V curve of In_2_O_3_/SnO_2_ sensor is nonlinear when the applied voltage drops in the reversely biased heterojunction, which is asymmetric for positive and negative voltages. The slightly rectifying behavior has to be ascribed to the different shape and crystalline state of the In_2_O_3_/SnO_2_ of the n-n homotype heterojunctions [35,48,49]. Electrochemical characteristics of In_2_O_3_/SnO_2_ sensors indicate the presence of *n*-*n* heterojunctions of two-type semiconducting oxides in In_2_O_3_/SnO_2_ composite hetero-nanofibers [48,50,51,52]. The sensing mechanism of homotype hetero-nanofibers sensing material needs further investigation. 

## 4. Conclusions

In_2_O_3_/SnO_2_ composite hetero-nanofibers were synthesized by a traditional electrospinning. The SnO_2_ nanofibers and In_2_O_3_ nanofibers exist simultaneously in the In_2_O_3_/SnO_2_ composite hetero-nanofibers. Each nanofiber is composed of small uniform nanocrystallites. The morphology and structure of all the nanofibers and SnO_2_ and In_2_O_3_ nanoparticles in same nanofibers are similar. Gas sensors were fabricated based on SnO_2_ nanofibers, In_2_O_3_ nanofibers, and In_2_O_3_/SnO_2_ composite hetero-nanofibers, respectively. The gas sensing properties of In_2_O_3_/SnO_2_ sensor were tested to six kinds of indoor volatile organic gases with gas concentration range of 0.5–50 ppm at the operating temperature of 275 °C. The gas sensor based on In_2_O_3_/SnO_2_ composite hetero-nanofibers exhibits the higher response to formaldehyde. The responses are 24.4 and 2.5 for formaldehyde concentrations of 50 ppm and 0.5 ppm, respectively. The response of In_2_O_3_/SnO_2_ composite hetero-nanofibers sensor was higher than that of SnO_2_ nanofibers or In_2_O_3_ nanofibers sensors, respectively. The gas sensing mechanism of the In_2_O_3_/SnO_2_ composite hetero-nanofibers sensor is analyzed. An *n-n* homotype heterojunction of In_2_O_3_/SnO_2_ may exist in the composite material, and the nanocrystallite boundary barrier of the homotype heterojunction may decrease when the composite materials meet formaldehyde, which leads to more electrons transfer and the increase of surface adsorption oxygen. As a result, the properties of In_2_O_3_/SnO_2_ composite hetero-nanofibers sensor are improved greatly. 

## Figures and Tables

**Table 1:** Element contents of In_2_O_3_/SnO_2_ composite hetero-nanofibers.

Element	Weight %	Atomic %
O	31.50	77.2
In	19.60	6.7
Sn	48.90	16.1
